# Synthesis of Graphene-Based Nanocomposites for Environmental Remediation Applications: A Review

**DOI:** 10.3390/molecules27196433

**Published:** 2022-09-29

**Authors:** Rohit Goyat, Yajvinder Saharan, Joginder Singh, Ahmad Umar, Sheikh Akbar

**Affiliations:** 1 Department of Chemistry, Maharishi Markandeshwar (Deemed to Be University), Mullana, Ambala 133203, Haryana, India; 2 Department of Chemistry, College of Science and Arts, and Promising Centre for Sensors and Electronic Devices (PCSED), Najran University, Najran 11001, Saudi Arabia; 3 Department of Materials Science and Engineering, The Ohio State University, Columbus, OH 43210, USA

**Keywords:** graphene, synthesis process, polymeric membranes, environmental remediation, composites

## Abstract

The term graphene was coined using the prefix “graph” taken from graphite and the suffix “-ene” for the C=C bond, by Boehm et al. in 1986. The synthesis of graphene can be done using various methods. The synthesized graphene was further oxidized to graphene oxide (GO) using different methods, to enhance its multitude of applications. Graphene oxide (GO) is the oxidized analogy of graphene, familiar as the only intermediate or precursor for obtaining the latter at a large scale. Graphene oxide has recently obtained enormous popularity in the energy, environment, sensor, and biomedical fields and has been handsomely exploited for water purification membranes. GO is a unique class of mechanically robust, ultrathin, high flux, high-selectivity, and fouling-resistant separation membranes that provide opportunities to advance water desalination technologies. The facile synthesis of GO membranes opens the doors for ideal next-generation membranes as cost-effective and sustainable alternative to long existing thin-film composite membranes for water purification applications. Many types of GO–metal oxide nanocomposites have been used to eradicate the problem of metal ions, halomethanes, other organic pollutants, and different colors from water bodies, making water fit for further use. Furthermore, to enhance the applications of GO/metal oxide nanocomposites, they were deposited on polymeric membranes for water purification due to their relatively low-cost, clear pore-forming mechanism and higher flexibility compared to inorganic membranes. Along with other applications, using these nanocomposites in the preparation of membranes not only resulted in excellent fouling resistance but also could be a possible solution to overcome the trade-off between water permeability and solute selectivity. Hence, a GO/metal oxide nanocomposite could improve overall performance, including antibacterial properties, strength, roughness, pore size, and the surface hydrophilicity of the membrane. In this review, we highlight the structure and synthesis of graphene, as well as graphene oxide, and its decoration with a polymeric membrane for further applications.

## 1. Introduction

**Figure 1:**
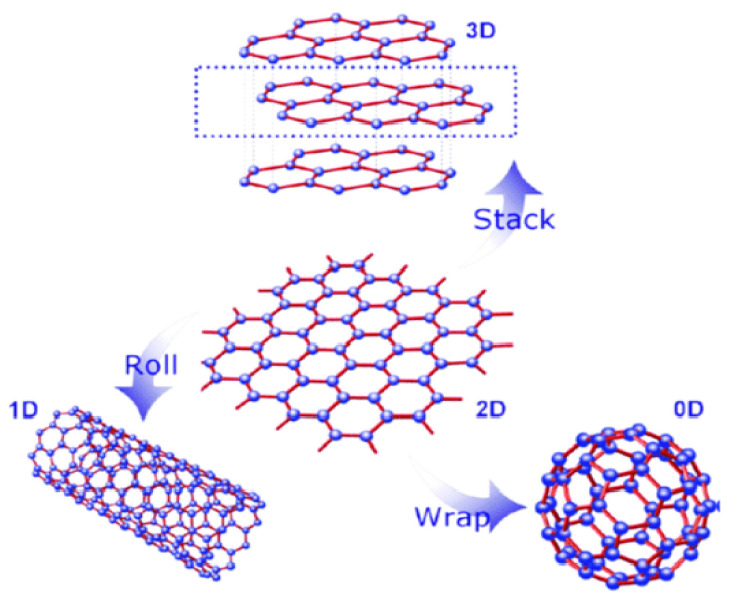
Structural representation of 2D graphene with different dimensions. [Reprinted with permission from ref. [28], Wan, X., Huang, Y., & Chen, Y. (2012). Focusing on energy and optoelectronic applications: a journey for graphene and graphene oxide at large scale. *Accounts of chemical research*, *45*(4), 598–607. Copyright © American Chemical Society].

Graphene is a purified form of graphite that recently gained enormous popularity in the energy [1,2,3], environment [4,5,6,7,8], membranes [1,7], sensor [9,10,11,12], and biomedical fields [13,14,15,16,17,18,19,20,21,22,23,24,25,26]. It is a sp^2^ hybridized, hexagonally arranged, chain of polycyclic aromatic hydrocarbon with a honeycomb crystal lattice [27]. It is the most recent element of carbon allotropes and is actually the basic building block of other important carbon allotropes, including 3D graphite, 1D carbon nanotubes (CNTs), and 0D fullerene (C60), as shown in Figure 1.

**Figure 2:**
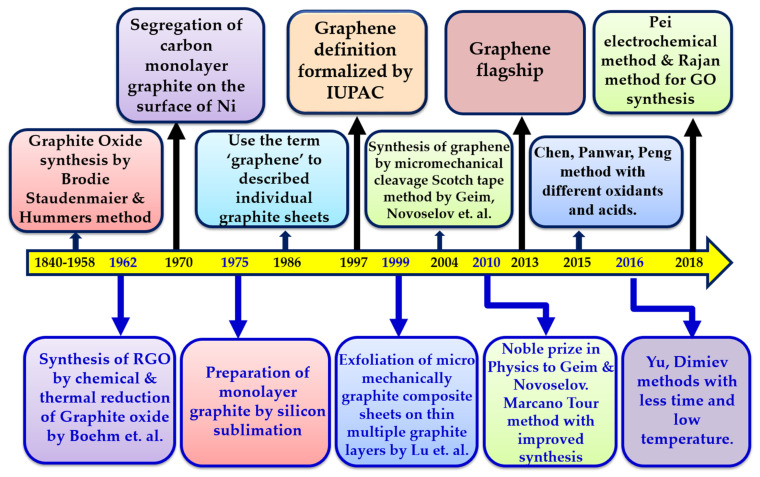
Schematic representation of a graphene timeline.

The name graphene was coined by Boehm in 1986 [1], taking the prefix “graph” from graphite and the suffix “-ene” for sp^2^ hybridized carbon, and was finally accepted by the International Union for Pure and Applied Chemistry in 1997 [29,30,31,32,33]. Furthermore, it became famous worldwide in 2004 when Geim and Novoselov obtained a single sheet of graphene on solid support, for which they were honored with the Nobel Prize in Physics in 2010 [34]. The main achievements of graphene in a timeline of history from 1840 to 2018 are shown in Figure 2.

## 2. General Methods of Graphene Synthesis

**Figure 3:**
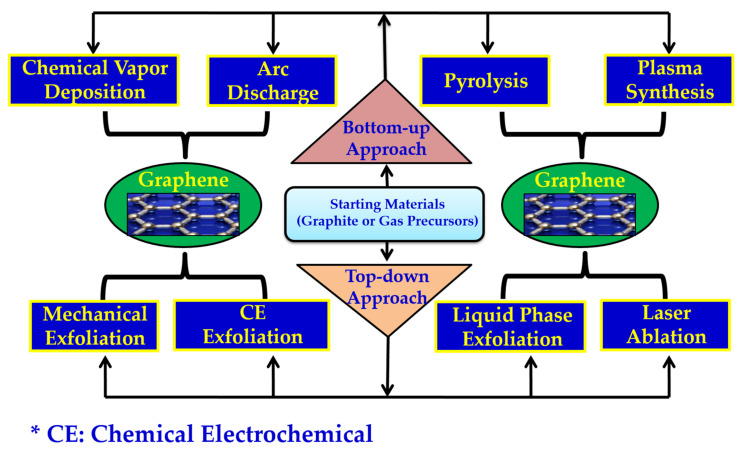
Schematic representation of the general methods for graphene synthesis.

Generally, graphene can be synthesized using two different routes, viz, bottom-up and top-down [33,35,36], as depicted in Figure 3.

### 2.1. Top-Down Method

In this method, graphite is exfoliated or converted into graphene [35,37] via mechanical, electrochemical exfoliation, laser ablation, and chemical/electrochemical fabrication. 

#### 2.1.1. Mechanical Exfoliation 

**Figure 4:**
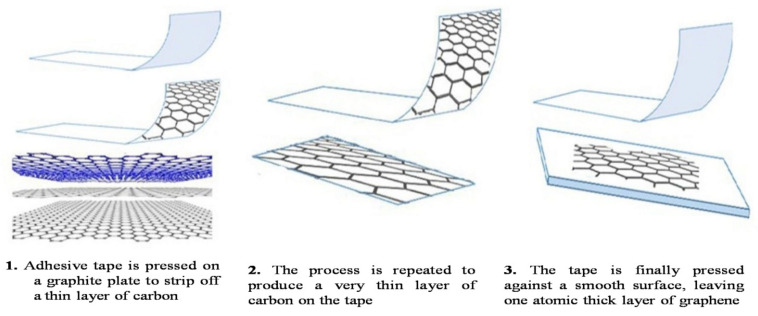
Mechanical exfoliation of graphene using the Scotch tape method [Reprinted with permission from ref. [39] Ibrahim, A.; Klopocinska, A.; Horvat, K.; Abdel Hamid, Z. Graphene-Based Nanocomposites: Synthesis, Mechanical Properties, and Characterizations. *Polymers* **2021**, *13*, 2869. https://doi.org/10.3390/polym13172869, Copyright © MDPI].

This method involves the stripping/peeling of layers of graphite using adhesive tape onto a SiO_2_ substrate. It was first invented by K. Novoselov and Andre Geim in 2004, and they were honored with a Nobel Prize for this invention [38]. Similarly, in 2017, Dasari et al. showed a micromechanical exfoliation of graphene sheets with adhesive Scotch tape [32]. Graphite was repeatedly peeled using adhesive tape until a single sheet of graphene was obtained, as depicted in Figure 4. Although this method is straightforward and is commonly used in laboratories, the obtained graphene has quite a low yield with few structural defects [38]. 

#### 2.1.2. Electrochemical Exfoliation

Electrochemical exfoliation is a technique in which the graphite as an electrode is exfoliated in an electrochemical cell under the effect of different electrolytes to give pure graphene. When a current is applied to the electrochemical cell, up to three layer of graphene sheets are exfoliated from the graphite, along with the formation of graphene intercalation compounds [40,41]. Many researchers tried different electrolytes for the exfoliation of graphite, resulting in improvements in size, thickness, and the chemical and electronic properties of graphene.

In other attempts by Parvez et al. in 2013, an electrolytic cell was prepared using graphite as an anode and platinum or other metal as cathode. The electrodes were immersed in an electrolyte solution of sulfuric acid with potential of +10 V for 10 min. The yield of this process was more than 60%, and the obtained graphene had multiple layers [42]. Similarly, Liu et al., 2013 used pencil graphite for both electrodes with 1.0 M H_3_PO_4_ as an electrolyte, and the obtained graphene was not homogeneous, with defects in thickness and size distribution [43]. Hence, the electrochemical exfoliation of graphite has gained concern as an easy and eco-friendly method to synthesize good-quality graphene.

#### 2.1.3. Liquid Phase Exfoliation 

**Figure 5:**
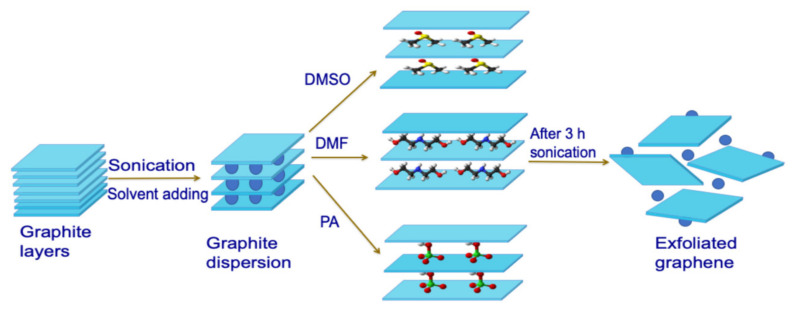
Schematic representation of the liquid phase exfoliation method [Reprinted with permission from ref. [48] Gürünlü, B.; Taşdelen-Yücedağ, Ç.; Bayramoğlu, M. Graphene Synthesis by Ultrasound Energy-Assisted Exfoliation of Graphite in Various Solvents. *Crystals* **2020**, *10*, 1037. https://doi.org/10.3390/cryst10111037, Copyright © MDPI].

Liquid phase exfoliation (LPE) is another top-down method in which sonication is performed for the exfoliation of the graphite into graphene layers, as depicted in Figure 5. In 2008, Hernandez et al. and Lotya et al. in 2009, used LPE sonication with different solvents, viz, acetic acid, sulfuric acid, and hydrogen peroxide, resulting in graphite converted to graphene [44,45,46]. The time of sonication was typically 50–55 min with a power supply of 280–500 W. In 2008, Li et al. confirmed that nanoribbons of graphene were produced using an LPE method wherein the width was less than 10 nm.

Further in 2009, Green and Hersam used sodium cholate as a surfactant for the exfoliation of graphite [47]. The advantage of LPE is that it is a reliable, scalable method for the synthesis of graphene, but high energy consumption and low yield are the main challenges that need to be addressed.

#### 2.1.4. Laser Ablation 

**Figure 6:**
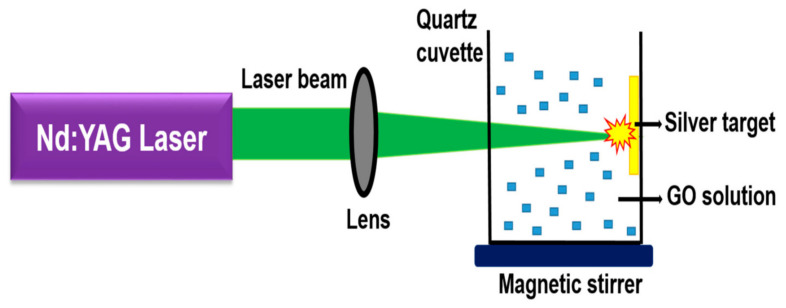
Schematic representation of the laser ablation method for graphene synthesis [Reprinted with permission from ref. [56] Nancy, P.; Jose, J.; Joy, N.; Valluvadasan, S.; Philip, R.; Antoine, R.; Thomas, S.; Kalarikkal, N. Fabrication of Silver-Decorated Graphene Oxide Nano-hybrids via Pulsed Laser Ablation with Excellent Antimicrobial and Optical Limiting Performance. *Nanomaterials* **2021**, *11*, 880. https://doi.org/10.3390/nano11040880, Copyright © MDPI].

In this technique, the laser erodes the carbon surface and produces graphene of the required quality. Several parameters, viz., laser beam repetition rate, wavelength, and pulse duration, must be checked during the synthesis process [49]. Secondly, the pressure of the gas in the background, the substrate distance, and the process temperature should also be in proper control [49,50,51]. Cappeli et al. opted for this technique in 2015 using silicon (Si) as a substrate [52]. He used a neodymium-doped yttrium aluminium garnet laser at different temperatures to obtain a high-quality graphene as shown in Figure 6. Further, in 2010 Koh et al. used ultra-short pulse laser technology with different substrates such as nickel, copper, cobalt, and iron for graphene synthesis [53]. The obtained graphene was of high quality with minimal size defects. Moreover, this process was quite eco-friendly with the ease of the experimental settings resulting in long-lasting graphene stability [54,55]. The noticeable disadvantages of this process were high energy inputs and the requirement for a much less laser-irradiating region for evaporating the target material.

### 2.2. Bottom-Up Method

This method is generally a self deposition or self-assembling process of nanoparticles carried out using four subtechniques, such as: arc discharge, chemical vapor deposition, pyrolysis, and plasma-enhanced chemical vapor deposition. In these subtechniques, the deposition of the graphite is carried out under controlled parameters like pressure, temperature, and flow rate [57]. The obtained graphene is of superior quality, having zero structural defects and possessing good electronic properties. However, the yield of the obtained graphene was rather low and could be used for limited applications. 

#### 2.2.1. Chemical Vapor Deposition (CVD)

**Figure 7:**
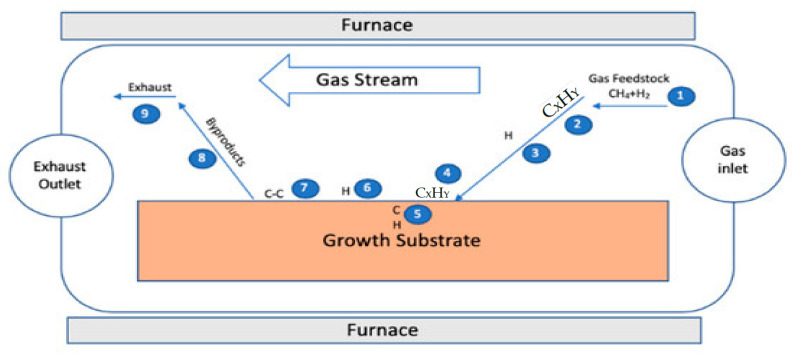
Schematic representation of the CVD method for graphene [Reprinted with permission from ref. [67], Saeed, M.; Alshammari, Y.; Majeed, S.A.; Al-Nasrallah, E. Chemical Vapour Deposition of Graphene Synthesis, Characterisation, and Applications: A Review. *Molecules* **2020**, *25*, 3856. https://doi.org/10.3390/molecules25173856, Copyright © MDPI].

In CVD, general equipment consists of tube furnace, gas flow, substrates, and tail gas treatment, as depicted in Figure 7. The commonly used substrates are from group B elements, which allow a low-energy pathway by forming intermediate compounds for the synthesis of graphene. The first row of d-block metals, viz, copper, cobalt, iron, and nickel, attracts huge interest due to their high availability and cost effectiveness [58]. The difference in the solubility of carbon with transition metals influences the growth quality of graphene [59]. Iron shows the highest carbon solubility, while copper has the lowest. For this reason, copper is a perfect metal to synthesize mono layer graphene, whereas, when both nickel and cobalt are used, multiple layers of graphene are often obtained. Graphene with a large surface area can be synthesized by exposing the precursors at extreme heat, wherein a copper or nickel substrate is placed at temperatures of 1000 °C in a reactor [60]. Furthermore, several scientists discussed the use of group B metals for the synthesis of graphene at large scale [60,61,62,63,64,65]. The quality of the substrate, temperature, and pressure provided on the surface of the substrate also regulates the synthesis of graphene in this process [66]. Due to the large number of interdependent parameters, the optimization process of the quality of graphene is typically very difficult in this process.

#### 2.2.2. Arc Discharge 

**Figure 8:**
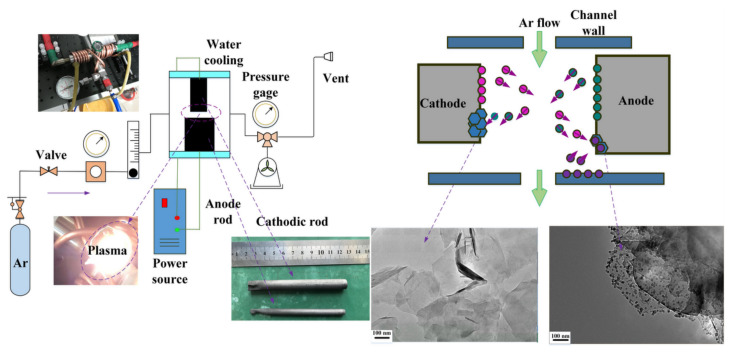
Schematic representation of the arc discharge method for the synthesis of graphene [Reprinted with permission from ref. [71], Tan, H.; Wang, D.; Guo, Y. A Strategy to Synthesize Multilayer Graphene in Arc-Discharge Plasma in a Semi-Opened Environment. *Materials* **2019**, *12*, 2279. https://doi.org/10.3390/ma12142279, Copyright © MDPI].

Krastchmer and Hoffman were the first to use the arc discharge method. In this method, an electric arc oven comprises two graphite electrodes with a steel chamber cooled with water, and further direct current arc voltage is applied across these two graphite electrodes immersed in an inert gas, as shown in Figure 8. Wu et al. in 2010 proposed the arc discharge method to synthesize graphene under the gaseous atmospheric conditions. He used a combination of hydrogen and nitrogen gases, to generate a graphene with good quality [68]. In comparison to chemical methods, the graphene produced had fewer structural defects and was easily dispersible in organic solvents, which enhanced its further applications. Other combination of gases, such as helium and carbon dioxide, were also tried, resulting in high-quality graphene production. Using the same process, the good quality bi- and tri-layers of graphene were reported in 2016 by Kim et al. [69]. In addition to this, in 2018, Cheng et al. combined vacuum arc discharge by using the CVD method for graphene synthesis [70]. Hence, arc discharge is an eco-friendly, cost-effective method that yields high-purity graphene.

#### 2.2.3. Plasma-Enhanced Chemical Vapor Deposition Synthesis (PECVD) 

The PECVD process is commonly designed to produce graphene from a hydrogen/methane gas mixture on copper and nickel samples. It is an additional method for the production of graphene that is similar to the thermal CVD process [71,72,73,74,75,76]. The process depends on the number of plasma sources, such as microwave (MW) [77], radio frequency [78], and direct current (dc) arc discharge [79]. Both copper and nickel are generally taken as the core substrates for PECVD graphene synthesis; yet, some other substrates have also been used [80,81]. The typical conditions for PECVD graphene synthesis on the metal substrate are methane in hydrogen (5–100%), with a substrate temperature in the range of 500 to 800 °C [81,82] and 900 W plasma power. The main advantage of this method is the low temperature and short time duration (<5 min) in comparison to thermal CVD.

#### 2.2.4. Pyrolysis

The pyrolysis or devolatilization process is the thermal decomposition of materials at high temperature in the atmosphere of inert gases. A change occurs in the chemical onfiguration of the starting material. To fabricate few-layer graphene, carbon atoms were synthesized on a metal surface. One of the familiar techniques to synthesize graphene is the thermal decomposition of silicon carbide (SiC) [83]. At elevated temperature, Si is desorbed, leaving behind carbon. The obtained graphene sheets have thickness up to 10 μm. The major advantages of this scheme are that it is cost-effective, providing for the simple fabrication of graphene. 

The advantages and disadvantages of the above-mentioned methods used for graphene synthesis are mentioned in Table 1 given below.

## 3. Graphene Oxide (GO)

**Figure 9:**
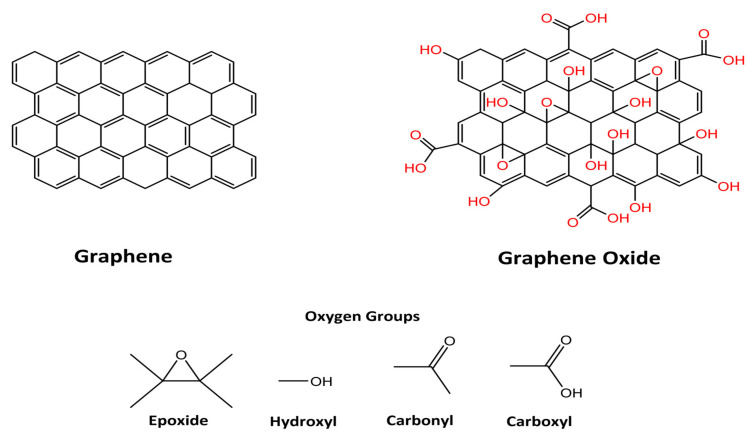
Schematic representation of the graphene and graphene oxide structures and graphene oxide oxygenated groups. [Reprinted with permission from ref. [87], Olean-Oliveira, A.; Oliveira Brito, G.A.; Cardoso, C.X.; Teixeira, M.F.S. Nanocomposite Materials Based on Electrochemically Synthesized Graphene Polymers: Molecular Architecture Strategies for Sensor Applications. *Chemosensors* **2021**, *9*, 149. https://doi.org/10.3390/chemosensors9060149, Copyright © MDPI].

In comparison to graphene, graphene oxide is considered a more versatile and advanced material. GO has a broad range of oxygen containing functional groups such as carboxyl, hydroxyl, epoxy, carbonyl, and keto groups on its surface, as shown in Figure 9 [84,85,86,87]. 

**Figure 10:**
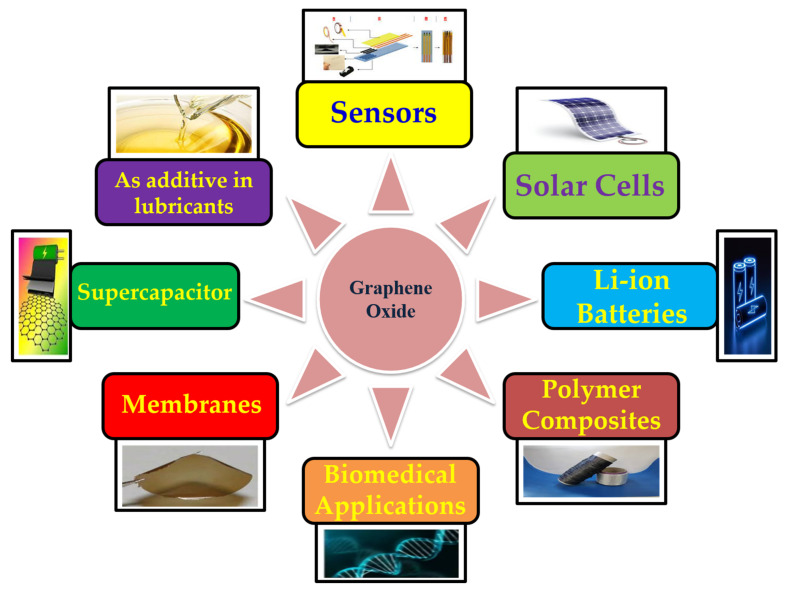
Schematic representation of the various applications of graphene oxide.

GO has shown great potential in a variety of fields by virtue of its high surface area [88], unique mechanical strength [89], and excellent optical and magnetic properties [90]. In comparison to other carbon-based nanomaterials, GO is considered a green oxidant, as it is enriched with oxygen-containing functional groups [91,92]. Further, GO has an aromatic scaffold, which acts as a template to anchor active species behaving as an organo-catalyst [93,94]. Hence, GO can replace conventional materials in a variety of applications in different fields as shown in Figure 10. 

### 3.1. Synthesis of GO

In 1840, German scientist Schafhacutl was given the first report on the synthesis of graphene oxide and graphite intercalated compounds [95]. For the very first time, he attempted to exfoliate graphite and tried to purify impure graphite “kish” from iron smelters [27]. To date, several methods, as shown in Table 2, have been proposed.

**Figure 11:**
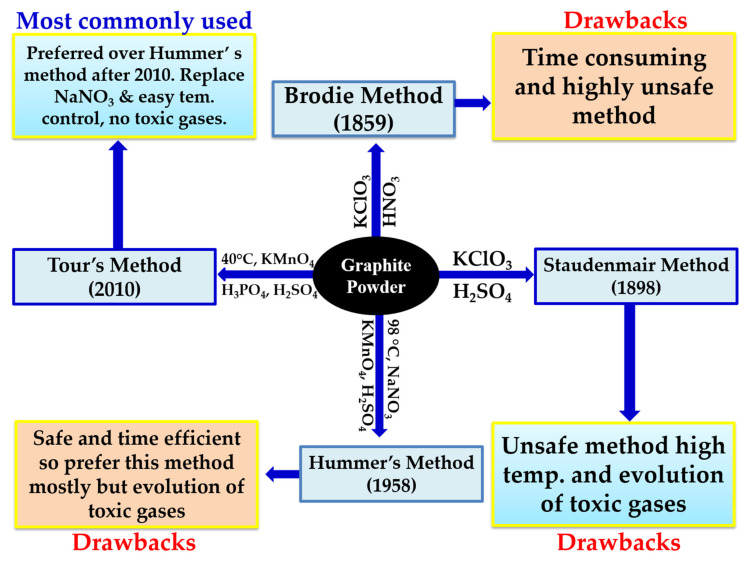
Schematic representation of the synthesis of graphene oxide with different methods.

The most preferred methods are Brodie [96], Staudenmaier [97], and Hummers [98], as shown in Figure 11. From these familiar methods, a number of variations have been derived to improve the overall yield and quality of the GO. In 1859, Brodie used graphite as the starting material for the synthesis of graphene oxide (GO). In his experimental work, he used KclO_4_ (strong oxidizing agent) along with nitric acid and heated the content at 60 °C for 3–4 days [96]. The GO obtained was soluble in pure or basic water. The chemical composition showed mainly carbon, oxygen, and hydrogen with the general formula C_11_H_4_O_5_. After nearly four decades, in 1898, Staudenmaier and Hoffmann modified Brodie’s method and trimmed down the reaction time of graphene oxide synthesis from 4 days to 2 days [97]. The nitric acid used in Brodie method was also replaced with sulfuric acid, which further reduced the liberation of toxic gases such as NO_2_ or N_2_O_4_.

In 1958, Hummer reduced the reaction time from 2 days to 12 h by using KmnO_4_ as the oxidizing agent instead of KclO_4_, followed by the addition of sodium nitrate, but the problem of toxic gases still remains a challenge [98]. Further, in 2010, at Rice University, Tour’s group [102] replaced sodium nitrate with phosphoric acid and increased the amount of KmnO_4_. This improvement made the process eco-friendly, as it completely stops the release of toxic gases such as NO_2_, N_2_O_4_ or ClO_2_, along with easy temperature control and better yield [102]. In addition to this, the GO suspension obtained was treated with hydrogen peroxide (H_2_O_2_) to eliminate all impurities due to permanganate and manganese dioxide.

Furthermore, the final color of the product GO varies from army green to light yellow, depending on the carbon-to-oxygen ratios [113], as depicted in Table 3.

#### 3.1.1. Post-Synthesis Treatment of GO

The post-synthesis treatment or workup of GO is a must, as the synthesized GO contains a noticeable amount of impurities, viz, the starting material (graphite), oxidizing agents, and the acids [114]. The workup of the graphene oxide could be performed via filtration and centrifugation techniques [114,115,116]. The common soluble contaminants, viz, ions of metal, sulfate, nitrate, phosphate, and manganese (IV) were removed by washing with a dilute HCL solution a number of times [114,115,116]. After each wash, GO was recuperated either by vacuum filtration or by centrifugation. Finally, the residues of HCl trapped inside GO were removed by washing with a sufficient quantity of de-ionized water [116,117,118,119,120]. Chen et al. carried out the final washing of GO with a HCl solution (5–10%) through filter paper supported on the funnel [117]. The key features of the process were that it offers high-quality GO totally free from sulfate, phosphate, manganese, and metal ions. Hirata et al. further improved the finishing washing step after centrifugation by giving the final wash with H_2_SO_4_ and H_2_O_2_ solutions [121].

#### 3.1.2. Effect of Various Temperatures on the Oxidation Level of GO

Various properties of GO, viz, electrical conductivity, band gap energy, transparency, optical properties, and surface charge are deeply influenced by the content of oxygen in carbonyl moieties present in GO after the oxidation of graphene [122,123,124]. These oxygen-containing functional groups act as excellent nucleation sites for the growth of inorganic materials over the surface of GO [125,126,127]. Therefore, to enhance the properties of GO sheets for various applications, it is required to control the oxidation of graphite to tune the amount of oxygen functional groups. This oxygen content on graphene can be controlled by the temperature maintained during the oxidation process of graphene [128,129,130]. This has been proven by the research carried out by Shin and co-worker in 2012 and Bannov et al. in 2014 [131,132,133]. Shin and co-worker prepared the GO sheets using the modified Hummers method, performed at different oxidation temperatures, as shown in Table 4. According to their procedure, the addition of cold concentrated sulfuric acid and potassium permanganate in a pre-oxidized graphite powder reaction mixture was stirred at 35 °C for two hours. This temperature was changed to 20 °C and 27 °C, respectively, for other samples [132,134]. From the elemental analysis, it was observed that more functional groups were formed during the oxidation process at higher oxidation temperatures. Further, these functional groups act as first-class nucleation sites for the expansion of inorganic materials like zinc oxide (ZnO), silica (SiO_2_), and titania (TiO_2_). In conclusion, it can be said that, to further enhance the properties of GO sheets for various applications, it is necessary to control the oxidation of graphite to tune the amount of oxygen functional groups [122,123,124,125,126,127,128]. 

### 3.2. Structural Aspects of GO 

**Figure 12:**
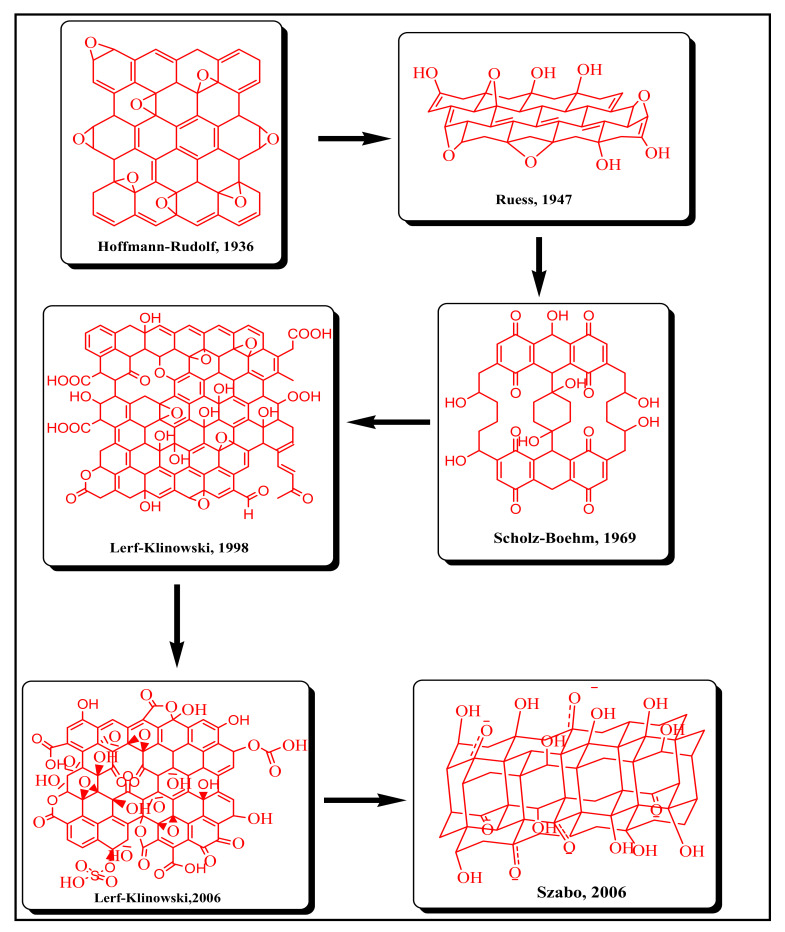
Schematic representation of the year-wise progress in proposed structures of graphene oxide [135,136,137,138,139,140,141,142].

Various structural models of GO, as shown in Figure 12, have been proposed and were refined over the years by the advancement of characterization techniques and technologies. The structural history of GO started in 1936, when Hofmann and Rudolf [135] proposed the first structure of GO in which epoxy groups were unsystematically spotted over the graphene sheets, and then in 1946, Ruess [136] restructured the Hofmann model by introducing hydroxyl moieties and the alternation of the basal plane structure from an sp^2^ to an sp^3^ hybridized carbon system. 

Scholz and Boehm in 1969 [137] proposed a GO structure that was less ordered, having C=C and periodically cleaved C-C bonds within the channeled carbon layers labeled with carbonyl and hydroxyl groups. Further, in 1994, Nakajima and Matsuo [138] presented a graphite intercalation compound (GIC) to look like a lattice framework. Adding to the history, in 1998, Lerf and Klinowski et al. (L–K model) [139,140] proposed a uniform carbon lattice framework GO structure with randomly distributed benzene rings having attached epoxides, carboxyl, and hydroxyl groups. Thereafter, in 2006, Szabó and coworkers [141] put forward a carboxylic-acid-free model comprising two distinct domains: a trans-linked cyclohexyl species interspersed with tertiary alcohols, 1,3-ethers, and a keto/quinoidal species corrugated network. Even closer to the present time, in 2018, Liu et al. [142] experimentally noticed oxygen bonding and evidenced the C=O bonds on the edge and plane of GO, confirming parts of earlier proposed models, especially the L–K model. 

Among the above-discussed models from 1936 to 2018, the L–K model has been accepted the most, due to good interpretability over the majority of experimental observations and the ease of further adaption and modification.

### 3.3. Characterization of GO

**Figure 13:**
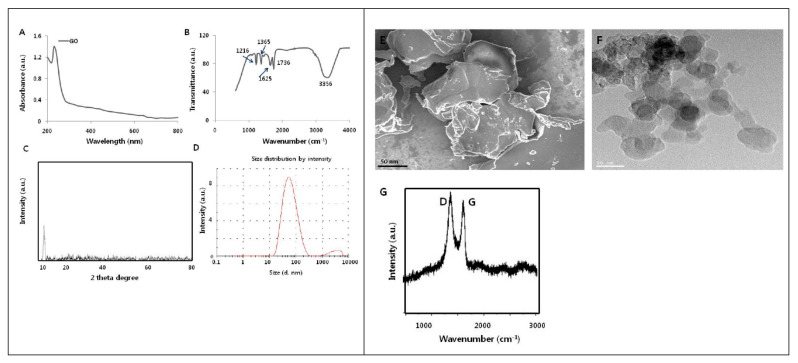
Schematic representation of the various characterizations of graphene oxide. (**A**) UV–visible spectrum, (**B**) FTIR spectrum, (**C**) X-ray diffraction images, (**D**) dynamic light-scattering analysis, (**E**) scanning electron microscopy, (**F**) transmission electron microscopy, (**G**) Raman spectrum [Reprinted with permission from ref. [164], Gurunathan, S.; Arsalan Iqbal, M.; Qasim, M.; Park, C.H.; Yoo, H.; Hwang, J.H.; Uhm, S.J.; Song, H.; Park, C.; Do, J.T.; Choi, Y.; Kim, J.-H.; Hong, K. Evaluation of Graphene Oxide Induced Cellular Toxicity and Transcriptome Analysis in Human Embryonic Kidney Cells. *Nanomaterials* **2019**, *9*, 969. https://doi.org/10.3390/nano9070969] Copyright © MDPI].

In order to authenticate the synthesis of GO and to analyze its chemical configuration, a range of characterization techniques have been employed by numerous research groups. For example, in order to achieve the information of size and surface morphology of graphene oxide, SEM, TEM, and AFM were used abroad [143,144,145,146,147]. With respect to the elemental analysis of graphene oxide, quantitative XPS, EDX, and inductively coupled plasma mass spectrometry (ICP-MS) were utilized generally [148,149,150,151,152,153,154,155,156]. Additionally, Raman spectra, XRD, and FTIR spectra are widely used to point out the graphene oxide chemical structure [156,157,158,159,160]. To get additional details about the properties of graphene oxide, TGA, and Zeta potential were also engaged by various research groups to evaluate its thermal stability and electrochemical property. More detailed explanations about these above-mentioned techniques are summarized in Table 5 and Figure 13.

**Table 5:** Various techniques for the characterization of GO.

Technique Used to Characterize Graphene Oxide	Information Obtained	Properties of Compound Observed	References
SEM	Lateral size distribution of GO sheets, showing the structural morphology of GO	Micromorphology and size of graphene oxide	[146]
TEM	Morphology of GO (wrinkles) and single-layered GO sheets.		[147]
AFM	Lateral size and thickness of GO sheets		[148]
TGA	Thermal stability of GO	Thermal stability	[149,150]
XPS	Quantitatively analyze the chemical composition of elements present in GO	Chemical structure of GO	[151,152,153,154,155,156]
			[157,158]
FTIR	Characteristic bands corresponding to carbonyl functional groups, confirmed the successful synthesis of GO		
XRD	Crystalline structures of the GO nanosheets and the inter-sheet distance of GO		[159,160]
Raman spectroscopy	Analyzing the chemical structure of GO combined with XPS, FTIR, XRD, ICP-MS.		[161,162]
UV spectroscopy	Help in structure identify	Presence of conjugated and non-bonding electrons	[163]

Abbreviations: SEM: scanning electron microscopy, TEM: transmission electron microscopy, XRD: X-ray crystallography, AFM: atomic force microscopy, TGA: thermogravimetric analysis, XPS: X-ray photoelectron spectroscopy, FTIR: Fourier transform infrared spectroscopy, UV: ultraviolet.

## 4. GO–Metal Oxide Nanocomposite Tailoring for Enhanced Water Purification Applications

Graphene oxide (GO) is no doubt a rising star material for nano-building and has shown great potential in membrane technology for water purification [164,165,166]. The properties of GO can be extra enhanced by modifications with adding a little sum of divalent alkaline earth metal ions bonded to the functional groups of GO layers [167]. These divalent metal ions act as a cross-linking building block between two adjacent carboxyl moieties of the GO layers and increase their solidity as well as stability. GO can also form composites when blended with carbon nanotubes, metal and their oxides, polymers, and some organic molecules, which work as spacers to prevent GOs restacking and helps in making the graphene material more porous [168,169,170,171,172,173].

**Figure 14:**
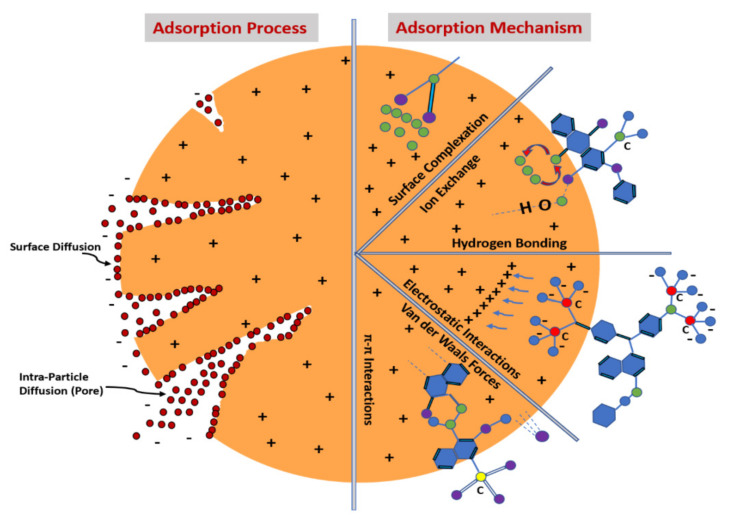
Schematic representation of the adsorption process and its mechanism for the adsorption of pollutants [Reprinted with permission from ref. [169], Hamad, H.N.; Idrus, S. Recent Developments in the Application of Bio-Waste-Derived Adsorbents for the Removal of Methylene Blue from Wastewater: A Review. *Polymers* **2022**, *14*, 783. https://doi.org/10.3390/polym14040783], Copyright © MDPI].

The purification ability of an adsorption process depends on the properties of the adsorbent used. Some functional groups, viz, -C=O, -COOH, -OH, -C-O-C on the graphene oxide surface make GO an excellent adsorbent. Further, due to the huge surface area, a large number of active binding sites and the electron-rich environment of GO nanocomposites have been successfully employed for the adsorption of various pollutants, including pesticides, heavy metals ions, different types of organic dyes, and other organic pollutants [167,168,169]. Figure 14 highlights the different interactions between active sites and the pollutant molecules [169]. 

In the present time, the fast development of industries even in the countryside causes the contamination of natural water reservoirs through the ejection of poisonous industrial by-products [164,165,166]. Therefore, the photocatalytic decomposition of industrial organic by-products is another encouraging technique to eradicate this problem. The word ‘photocatalytic decomposition’ is referred towards the complete conversion of harmful and less-likely-degradable contaminants into harmless compounds. Heterogeneous photocatalysis involves organic synthesis, water-splitting, photo-reduction, hydrogen transfer, disinfection, water detoxification, gaseous pollutant eradication, etc. The different metal oxide nanoparticles utilized along with GO in the last two to three decades are of silver oxide, titanium dioxide, zinc oxide, copper oxide, aluminum oxide, iron oxide, and zirconium dioxide have also been reported [174,175]. We have reviewed some features of GO as an adsorbent for dyes, metal ions, antibacterial activities, and environmental applications as shown in Table 6. In gas detection activities, graphene-based nanomaterials have been extensively investigated because of their high sensitivity toward various gaseous species. Few-layered hydrophilic sheets of graphene oxide manifest amazing adsorption behavior towards miscellaneous harmful gases such as CO_2_, CO, NO_2_, and NH_3_ [176].

The combination of silver–graphene oxide (Ag/GO) nanocomposites has been reported as an excellent antibacterial agent for water disinfection. According to Sun. et al., the Ag/GO nanocomposite has been further developed for an antibacterial water purification membrane [177]. The graphene oxide sheets were used as an adsorbent for the rapid uptake of four various pesticides from water samples and might be used as a good antioxidant and an antibacterial agent [178]. In addition to this, the SiO_2_/GO nanocomposite showed a major improvement in terms of water flux, pollutant rejection, and antifouling tendency in membranes [179]. Moreover, the combination of TiO_2_/GO also shows a vast performance in different aspects such as hydrophilicity, water permeability, and fouling resistance [180]. Besides, the synthesis of the ZnO–GO combination has been shown to have enhanced photocatalytic and antimicrobial activity. ZnO nanoparticles can also be used as a water-body restoration material, and it can diminish up to 97% of MB dye under ultraviolet radiation conditions.

**Table 6:** Some recently published studies on GO–metal oxide nanocomposites and major pollutant trapped for environmental remediation.

GO/Metal Oxide Nanocomposites	Main Pollutant Trapped	Achievements	Reference
GO–silver oxide	Cyclohexane	By using GO–Ag composities as photocatalysts, 37.0% conversion and 94.0% selectivity of cyclohexane to cyclohexanol was achieved.	[181]
Graphene-supported Fe–Mg oxide composite	Arsenic heavy metal ions	The prepared composite exhibited the significant fast adsorption of arsenic with exceptional durability and recyclability.	[182]
GO/Fe_3_O_4_	Methylene blue and rhodamine B dyes	The dye removal rate for methylene blue was nearly 100%, while for rhodamine B, it was about 90%.	[183]
GO–MnFe_2_O_4_	Pb(II), As(III), and As(V) heavy metal ions	The exceptional adsorption property was due to a combination of the unique layered nature (allowing the maximum surface area) of the hybrid system and the good adsorption capabilities of nanoparticles.	[184]
GO–ZrO(OH)_2_	As(III) and As(V) heavy metal ions	The GO–ZrO(OH)_2_ nanocomposite showed a high adsorption capacity in a wide pH range, and the monolayer adsorption amounts were 95.15 and 84.89 mg/g for As(III) and As(V).	[185]
GO–iron oxides	Pb(II) heavy metal ion	The GO–iron oxide nanocomposite acts as a good adsorbent for Pb(II).	[186]
GO–TiO_2_	Zn^2+^, Cd^2+^, and Pb^2+^ heavy metal ions	The various and dense oxygenated moieties on the GO surface enhanced its capacity to absorb heavy metal ions.	[187]
Graphene–ZnO	Methyl orange dye	The maximum photocatalytic degradation efficiency of methyl orange was 97.1% and 98.6% under UV and sunlight, respectively.	[188]
ZnO–GO/nanocellulose	Ciprofloxacin organic pollutant	The synthesized nanocomposite exhibited enhanced adsorption and photocatalytic performance against ciprofloxacin.	[189]
GO/goethite	Tylosin organic pollutant	The degradation efficiency of the antibiotic by the synthesized composite was 84% after 120 min.	[190]
CuO–CeO_2_/GO	Methyl orange dye	The nanocomposite showed better catalytic activity than pure CuO and CuO/GO in the presence of H_2_O_2_ under visible light irradiation.	[191]

### Inclusion of GO–Metal Oxide Nanocomposites into Polymeric Membranes for Enhanced Performance and Application in Different Fields

**Figure 15:**
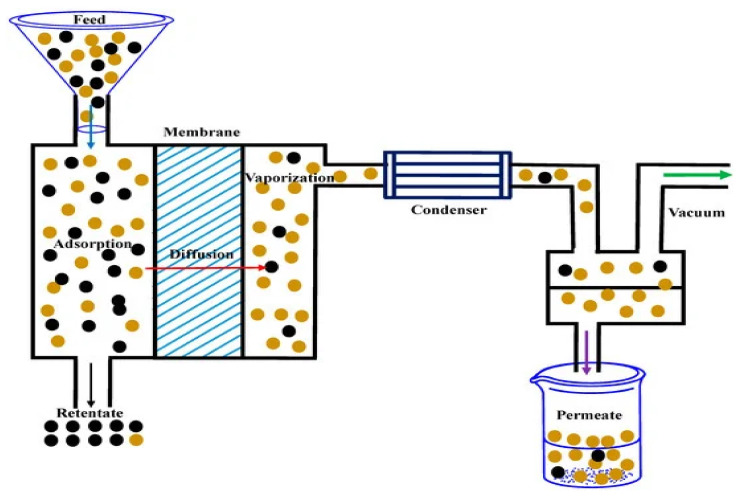
Schematic representation of the basic purification setup with a membrane [Reprinted with permission from ref. [196], Roy, S.; Singha, N.R. Polymeric Nanocomposite Membranes for Next Generation Pervaporation Process: Strategies, Challenges and Future Prospects. *Membranes* **2017**, *7*,53. https://doi.org/10.3390/membranes7030053], Copyright © MDPI].

Over the last few decades, enormous efforts were made to synthesize different types of membranes that could be further employed for a number of applications, viz, drinking-water filtration, use in food [192], the beverage and textile industries [193], petroleum refining [194], paint, and adhesive and solvent recovery stations [195], as shown in Figure 15.

**Figure 16:**
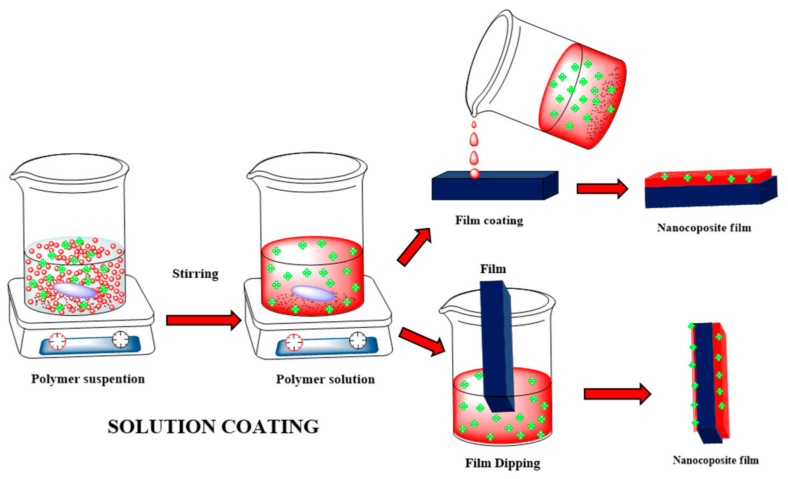
Schematic representation of the fabrication of a polymeric membrane [Reprinted with permission from ref. [196], Roy, S.; Singha, N.R. Polymeric Nanocomposite Membranes for Next Generation Pervaporation Process: Strategies, Challenges and Future Prospects. *Membranes* **2017**, *7*, 53. https://doi.org/10.3390/membranes7030053], Copyright © MDPI].

Despite the good success in the membrane filtration technology, some difficulties and drawbacks [197,198,199,200,201] still need to be studied and discussed. The main drawbacks that limit their application at large scale are membrane fouling [200], membrane choking, and, finally, membrane crumbling. Among these, membrane fouling is the real beginning of the problem [200]. The invasion of bacteria and, further, their colonization on the membrane surface leads to the formation of a microbial biofilm [198], clogging the membrane pores and blocking and restricting the water flow through it [199]. Furthermore, once the microbial biofilm is formed, it becomes quite difficult to remove it. As a result, a large amount of cleaning agents are used, which increase the operation and maintenance costs [198,199,200]. Numerous research groups have tried different technologies to fabricate the membrane, viz; interfacial polymerization, track-etching, coating, stretching, phase inversion, and electro-spinning for the modification and improvement of the membrane surfaces, but it still requires a lots of improvement [202,203,204,205]. Some common techniques used to fabricate the membranes were shown in Figure 16.

Further, various types of polymers are tried as a core material, along with organic solvents and inorganic metal oxides, as shown in Table 7, to remove the above-mentioned limitations. Polymers, such as polyvinylidine fluoride, polysulfone, polyethersulfone, polyacrylonitrile, polypropylene, and polytetrafluoroethylene, offer a great design with high flexibility and stability to the membrane [206,207,208,209,210]. Furthermore, to improve the porosity, antibacterial, and anti-fungal activity, other additives such as metal oxide/graphene oxide nanocomposites and organic solvents were incooperated in membrane synthesis by numerous research groups [209,210,211,212].

**Figure 17:**
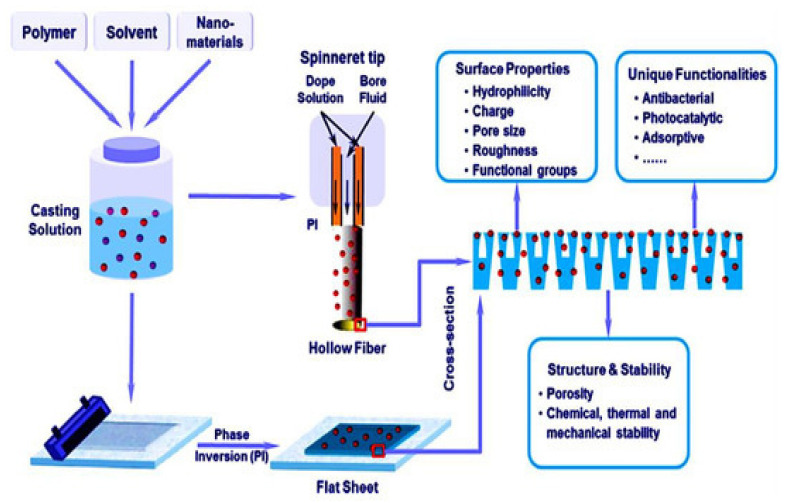
Schematic representation of the fabrication of a polymeric nanocomposite membrane, along with its surface properties [Reprinted with permission from ref. [87], Kausar, A.; Bocchetta, P. Polymer/Graphene Nanocomposite Membranes: Status and Emerging Prospects. *J. Compos. Sci.* **2022**, *6*, 76. https://doi.org/10.3390/jcs6030076, Copyright © MDPI].

Nanoparticles may be either coated onto the membrane surface or dispersed in the polymer solution before membrane casting, as shown in Figure 16. Dispersing the GO–metal oxide nanocomposites into the polymer generally forms these composite membranes that are a suitable tool to improve the performance, such as permeability and selectivity, of polymeric membranes, due to changes in the surface properties of membranes, influencing the separation performance, excellent rejection of pollutants, and better antifouling behavior, as shown in Figure 17 [208,209,210,211,212,213,214,215,216].

**Table 7:** Different polymeric membranes decorated with metal oxide nanocomposites.

Nanoparticle Used in Membrane	Membrane Type	Application	Polymer Used for Membrane	Reference
ZnO	MF	Treatment of synthetic wastewater	PVDF	[217]
		Removal of copper ions from water		[218]
		Removal of COD from wastewater		[219]
	UF	Removal of HA	PES, PSF	[220,221]
		Removal of salts	PA	[222]
		Evaluation of antifouling properties in composite membranes for water treatment. Mixture model: BSA	PVDF	[223]
		Removal of pollutants sodium alginate, BSA, and humic acid (HA)	PES	[224]
		Evaluation of antifouling properties in composite membranes for water treatment. Mixture model: BSA	PES	[225]
		Evaluation of antifouling properties in composite membranes for water treatment. Mixture model: BSA	PVA	[226]
	NF	Removal of HA	PES	[227]
		Water filteration	PVP	[228]
		Removal of inorganic salts and HA	PVDF	[229]
	RO	Removal of salt, bivalent ions (Ca^2+^ SO_4_^2-^ and Mg^2+^), monovalent ions (Cl^-^ and Na^+^), and bacterial retention	PA	[230,231,232]
	FO	Removal of salts, desalination	PVDF	[230,231]
GO	MF	Treatment of effluents with high-dye content and water filtration	PSF, PVDF	[233,234]
	UF	Treatment of distillery effluent	PES	[235]
		Natural organic matter removal	PA, PVDF	[236]
	NF	Evaluation of dye-removal capacity for water treatment	PES	[237]
	RO	Desalination: Salt removal (NaCl, CaCl_2_, and Na_2_SO_4_)	PSF	[238,239]
	FO	Possible prospect for the desalination of sea water	PA	[240]
Graphene	UF	Wastewater treatment	PSF	[241]
	NF	Water purification	PVDF	[242]
AgNO_3_	UF	Reduction of the microbial load of raw milk during the concentration process by the UF process	PES	[243]
		Evaluation of antifouling properties in composite membranes for water treatment. Mixture model: BSA	PSF	[244]
AgNPs	UF	Evaluation of antifouling and antibacterial properties in composite membranes for water treatment. Model bacteria: E. coli	PES, PSF, CA	[244,245]
AgNO_3_	RO	Evaluation of antibacterial properties and the removal of salt (NaCl). Model bacteria: E. coli and Bacillus subtilis	PA/PSF/PET	[246,247]
CuNPs	UF	Treatment of wastewater (sludge filtration) and the evaluation of antifouling properties in composite membranes for water treatment. Mixture model: BSA	PES	[248]
	RO	Evaluation of antibacterial properties in composite membranes for water treatment and the removal of salt (NaCl). Model bacteria: E. coli, P. aeruginosa, and S. aureus.	PA	[248]
TiO_2_-NPs	NF	Wastewater treatment application	PES	[249]
	UF	Evaluation of antifouling properties in composite membranes for water treatment. Mixture model: BSA, PEG, and MgSO_4_	PVDF	[250]
		Evaluation of UV-cleaning properties and antifouling properties. Mixture model: red dye and BSA	PA	[251]

Abbreviations: BSA—bovine serum albumin, CA—cellulose acetate, HA—hummic acid, PA—polyamide, PAA—poly(acrylic acid), PAI—poly(amide-imide), PAN—polyacrylonitrile, PEI—polyethyleneimine, PE—polyethylene, PEG—polyethylene glycol, PSF—polysulfone, PES—polyethersulfone, PVA—polyvinyl alcohol, PVDF—polyvinylidine fluoride, PVP—polyvinylpyrrolidone, PVC—polyvinyl chloride, PP—polypropylene, NF—nanofiltration, RO—reverse osmosis, UF—ultrafiltration, ZnO—zinc oxide, GO—graphene oxide, MF—microfiltration.

## 5. Challenges and Futuristic Aspects

The graphene-oxide-based nanomaterial, along with metal oxide nanocomposites, deposited on polymeric membranes have achieved excellent appreciation as a water purifier, but there are still few drawbacks and challenges that confine their use at a large scale.

Various routes of GO synthesis have been discussed in the research, and each route has given GO that is good quality-wise, but the overall yield is low. An improvement in the overall yield is a major concern. It needs to be addressed sooner, if we want to use GO in large-scale applications.

The second challenge is the aggregation of the GO–metal oxide nanomaterials on the membrane surfaces, which diminishes the active surface area, the porosity, and the overall performance of the membrane. Over the last two decade or so, many research [197,198,199,200,201,202] groups have made attempts to remove this challenge by making alterations in the synthesis of graphene oxide–metal oxide nanomaterial and decorated it on the polymeric membranes with different methods [201,202,203,204,205].

The third and the most important challenge is related to membrane strength, membrane-wetting, and membrane-fouling due to colloids and particles present in the feed flow, which tends to significantly reduce membrane performance, increase operating costs, and shorten membrane life.

## 6. Conclusions

In recent years, many research groups are paying attention towards graphene because of its sole physicochemical properties, viz, high tensile strength, better electrical and thermal conductivity, fast carrier mobility, elasticity, and about 97% optical transparency. In this review article, a brief account on the structure, properties, synthesis, characterizations, and applications of graphene, graphene oxide, and GO–metal oxide-decorated polymeric membranes are discussed. Since its discovery in 2004, graphene has resulted in a wide range of applications in various fields such as solar cells, supercapacitors, sensors, batteries, and water-purification technologies. In addition, the presence of an abundance of oxygenated moieties on the GO nanoparticles imparts a high negative charge density over the GO surface and improves the adsorption quality. The addition of the graphene-based materials in the polymeric membrane-based water-purification processes enhanced the positive impact on the hydrophilicity and the antifouling and antibacterial properties of the membranes. Furthermore, GO–metal oxide nanocomposites with increased antibacterial effects and low toxicity can be employed efficiently as disinfection agents in the surface coatings on numerous membranes to effectively suppress bacterial growth.

The aim of this review was to study the development of a novel high-tech membrane using a polymer decorated with a GO–metal nanocomposite to improve the overall membrane performance, including antibacterial properties, antifouling, porosity, and the surface hydrophilicity of the membrane.

## Figures and Tables

**Table 1:** Advantages and disadvantages of various methods used to synthesize graphene.

Top Down Method					
S. No	Methods	Thickness of Graphene Obtained	Advantage	Disadvantage	Reference
1	Micromechanical exfoliation	Single layer of graphene	Simple method with the formation of large size layers of graphene	Low yield	[32,33,34,35,36,37,38]
2	Electrochemical exfoliation	Single and few layers of graphene formed	High yield and quick process	Having structural defects and workup is expensive.	[40,41,42,43]
3	Liquid phase exfoliation	Mostly single layers of graphene obtained	Reliable and scalable method with the high exfoliation of graphite	Involves the use of hazardous chemical (chloro sulfonic acid) and the removal of used acid in the process is costly	[45,46,47]
4	Laser ablation	Single, bi, and multiple layers of graphene	Rapid, simple, and eco-friendly process with high-quality graphene.	Small laser-irradiating area for evaporating the target material	[49,50,51,52,53,54,55]
**Bottom Up Method**					
**S. No**	**Techniques**	**Thickness**	**Advantage**	**Disadvantage**	**Reference**
1	CVD	Mono and few-layer graphene sheets	Large size sheets of graphene obtained	Difficult to control numerous parameters	[61,62,63,64,65,66]
2	Arc discharge	Single, bi, and few layers of graphene	Cost-effective method with high-quality product	Requires a gaseous atmosphere, and the product contains structural defects	[68,69,70]
3	Plasma enhanced chemical vapor deposition	Bi and tri layers of graphene	Low temperature and less duration with high production	Requires high plasma power and different substrates	[74,75,76,77,78,79,80,81]
4	Pyrolysis	Few-layer graphene	Requires low cost and the high quality of graphene produced	Method used on a small scale	[83]

**Table 2:** List of different methods used to synthesize graphene oxide.

Methods	Year	Starting Material	Different Oxidants Used	Reaction Time for GO Synthesis	Temperature °C	Features	References
Brodie	1859	Graphite	KclO_3_, HNO_3_	3–4 days	60	First attempt to synthesize GO	[96]
Staudenmaier	1898	Graphite	KclO_3_, H_2_SO_4_, HNO_3_	96 h	Room temperature	Improved efficiency	[97]
Hummers	1958	Graphite	KmnO_4_, H_2_SO_4_, NaNO_3_	<2 h	<20–35–98	Water-free, less than 2 h of reaction time	[98]
Fu	2005	Graphite	KmnO_4_, H_2_SO_4_, NaNO_3_	<2 h	35	Validation of NaNO_3_	[99]
Shen	2009	Graphite	Benzoyl peroxide	10 min	110	Fast and non-acidic	[100]
Su	2009	Graphite	KmnO_4_, H_2_SO_4_	4 h	Room temperature	Large-size GO sheets formed	[101]
Marcano and Tour	2010 & 2018	Graphite	KmnO_4_, H_3_PO_4_, H_2_SO_4_	12 h	50	Eco-friendly resulting in a high yield	[102]
Sun	2013	Graphite	KmnO_4_, H_2_SO_4_	1.5 h	Room temperature-90	High-yield and safe method	[103]
Eigler	2013	Graphite	KmnO_4_, NaNO_3_, H_2_SO_4_	16 h	10	High-quality GO produced	[104]
Chen	2015	Graphite	KmnO_4_, H_2_SO_4_	<1 h	40–95	High-yield product	[105]
Panwar	2015	Graphite	H_2_SO_4_, H3PO_4_, KmnO_4_, HNO_3_	3 h	50	Three component acids and high-yield product	[106]
Peng	2015	Graphite	K_2_FeO_4_, H_2_SO_4_	1 h	Room temperature	Results in a high-yield and eco-friendly method	[107]
Rosillo-Lopez	2016	Graphite	HNO_3_	20 h	Room temperature	Nano-sized GO obtained	[108]
Yu	2016	Graphite	K_2_FeO_4_, KmnO_4_ H_2_SO_4_, H_3_BO_3_ (NH_4_)_2_S_2_O_8_	5 h	<5–35–95	Low manganite impurities and high yield obtained	[109]
Dimiev	2016	Graphite	98% H_2_SO_4_, fuming H_2_SO_4_	3–4 h	Room temperature	25 nm thick and ~100%conversion rate	[110]
Pei	2018	Graphite foil	H_2_SO_4_	<5 min	Room temperature	High efficiency	[111]
Ranjan	2018	Graphite	H_2_SO_4_, H_3_PO_4_, KmnO_4_	<24 h	<RT-35–95	Cooled exothermal reaction to make the process safe	[112]

**Table 3:** Effect of acid concentration, reaction temperature, reaction time, and the quantity of the oxidizing agent on the oxidation of graphene [113].

S. No.	Source of Carbon	H_2_SO_4_ (in mL)	Other Ingredients	Temp. (in °C)	Time (in h)	C:O	Colour of GO Obtained
1	Graphite	15.0	1.0 g Na_2_Cr_2_O_7_	30	72	16:1	Black
2	Graphite	15.0	4.0 g Na_2_Cr_2_O_7_	30	72	3.4:1	Black
3	Graphite	15.0	15.0 mL 70% HNO_3_ 3.0 g KmnO_4_,	30	24	--	Black
4	Graphite	20.0	11.0 g KclO_3_, 10.0 mL 70% HNO_3_	0–60	33	3.1:1	Midnight green
5	Graphite	30.0	3.0 g KmnO_4_,1.0 g NaNO_3_	30	2	3.0:1	Bluish green
6	Graphite	30.0	3.0 g KmnO_4_,1.0 g NaNO_3_	45	1	--	Green
7	Graphite	22.5	3.0 g KmnO_4_,1.0 g NaNO_3_	45	1	--	Brittle yellow
8	Graphite	22.5	3.0 g KmnO_4_,0.5 g NaNO_3_	45	1	--	Yellow
9	Graphite	22.5	3.0 g KmnO_4_,0.5 g NaNO_3_	45	0.5	2.3:1	Yellow
10	Graphite	22.5	3.0 g KmnO_4_,0.5 g NaNO_3_	35	0.5	2.05:1	Bright yellow
11	Graphite	22.5	3.0 g KmnO_4_, 1.0 g fuming HNO_3_	35	1	--	Bright yellow
12	Graphite	22.5	3.0 g KmnO_4_, 1.0 g BaNO_3_	45	2	--	Light green

**Table 4:** Analysis of the elements present in different samples at various temperatures [131].

Elements Present in GO (Weight %)	Sample 1	Sample 2	Sample 3
	Temperature 35 °C	Temperature 27 °C	Temperature 20 °C
Carbon	44.09	45.51	44.55
Oxygen	49.92	48.93	47.16
Hydrogen	3.30	2.96	3.02
Atomic ratio of carbon and oxygen	1.18	1.24	1.26
